# Neurological soft signs in borderline personality disorder and schizophrenia

**DOI:** 10.1186/s40479-025-00282-7

**Published:** 2025-03-17

**Authors:** Marie-Luise Otte, Mike M. Schmitgen, Nadine D. Wolf, Katharina M. Kubera, Yunus Balcik, Chantal Tech, Mert Koc, Yéléna Le Prieult, Fabio Sambataro, Geva A. Brandt, Stefan Fritze, Dusan Hirjak, Robert Christian Wolf

**Affiliations:** 1 Department of General Psychiatry, Center for Psychosocial Medicine, Heidelberg University, Vosstrasse 4, 69115 Heidelberg, Germany; 2 Department of Neuroscience (DNS), University of Padova, Padova, Italy; 3 Department of Psychiatry and Psychotherapy, Medical Faculty Mannheim, Central Institute of Mental Health, Heidelberg University, Mannheim, Germany; 4 German Center for Mental Health (DZPG), partner site Mannheim/Heidelberg/Ulm (ZIHUb), Germany

**Keywords:** Neurological soft signs, Borderline personality disorder, Schizophrenia, Childhood trauma, Treatment outcome

## Abstract

**Background:**

Neurological soft signs (NSS) are subtle sensorimotor abnormalities that have been observed in various mental disorders with neurodevelopmental origin. While NSS have been extensively examined in patients with schizophrenia (SZ), preliminary evidence also suggests that NSS are also present in patients with borderline personality disorder (BPD). However, a transdiagnostic examination of the severity of NSS in BPD compared to SZ is still lacking.

**Methods:**

Here, NSS were examined with the Heidelberg NSS scale (HNSS) in three groups of female subjects: BPD (*n* = 45), SZ (*n* = 30) and healthy controls (HC) (*n* = 32). Multivariate analysis of variance (MANOVA) was conducted jointly for BPD, SZ, and HC and HNSS subscores. Post hoc tests were performed using linear discriminant analysis (LDA). In the BPD group, partial Spearman correlations (with age and medication as covariates) were performed between NSS scores and depressive symptoms (HAMD-21), impulsivity (BIS-11), dissociative symptoms (DTS), childhood trauma (CTQ), and borderline symptoms (BSL-23).

**Results:**

BPD showed significantly higher NSS levels compared to HCs. For the BPD, significant associations between NSS and childhood trauma and depressive symptoms were found. MANOVA showed a significant group difference, LDA differentiated between HC, and patients with SZ and BPD, but not between the patient groups.

**Conclusions:**

Patients with BPD have significantly higher NSS levels than HC. NSS in BPD showed significant associations with childhood trauma, supporting a “two-hit” model. Importantly, patients with BPD and SZ may show similar NSS patterns, suggesting that sensorimotor dysfunction is a transdiagnostic phenomenon.

## Introduction

Neurological soft signs (NSS) are subtle sensorimotor abnormalities which comprise difficulties in sensory integration, motor coordination, inhibition, and sequencing of complex motor tasks [1]. In contrast to clinically apparent “hard signs”, e.g., clearly observable deficits in the arm holding test, NSS cannot be attributed to a specific brain lesion. Rather, at least in healthy people, NSS are thought to reflect the neurodevelopmental variation of the sensorimotor system and its coupling with non-sensorimotor functional networks [2, 3]. As a result of ongoing brain maturation, NSS levels decrease during childhood [4, 5], and various degrees of NSS persistence can be detected in healthy adults, being mostly stable over time. However, in the elderly, the NSS increase due to physiological aging processes, while in some cases, increasing NSS levels over time may also be indicative of neurodegenerative diseases [6, 7]. The vast majority of NSS research in mental disorders has been performed in schizophrenia (SZ) [8, 9]. Further, first-degree relatives of patients with SZ, including monozygotic twins, show higher NSS levels than healthy controls (HC) [10, 11] supporting NSS as a putative sensorimotor risk marker. NSS are found in antipsychotic-naïve patients with SZ in the first psychotic episode, as well as in people at risk of developing the disorder [12] and might predict the transition into manifest disorder [13]. In multiple episode SZ, NSS may show a more heterogeneous pattern over time, being frequently associated with negative symptoms and cognitive dysfunction [5, 9, 14]. More recently, NSS integrative functions were found to predict auditory verbal hallucinations (AVH) in SZ, supporting the notion of a close interrelationship between sensorimotor and perceptual abnormalities [15].

Beyond SZ, the extant research also suggests associations between NSS, affective and neurodevelopmental disorders such as autism or attention-deficit/hyperactivity disorder [9, 16–18], supporting the notion of a transdiagnostic neural signature of genuine sensorimotor system dysfunction that may be closely related to brain maturation. In this regard, it is noteworthy that increased NSS levels were also reported in individuals with personality disorders, particularly in borderline personality disorder (BPD) [19, 20]. Preliminary evidence suggests significantly increased NSS levels in BPD patients compared to HC, particularly considering the motor coordination and sensory integration domains [19, 21–24]. However, the significance of these findings is unclear at present. Some authors have argued in favor of a distinct BPD “psychomotor endophenotype” [19]. Other researchers suggested a close interrelationship between impulsivity and NSS in BPD [19, 23], whereas a more recent study highlighted a significant association between overall BPD severity and NSS [23].

To fill the extant gap of knowledge, this study has four major goals: (1) Investigating, if NSS levels are significantly different between BPD patients and HC, particularly with respect to motor coordination and sensorimotor integrative functions. (2) Exploring associations between NSS and BPD overall symptom load, distinct symptom dimensions (e.g., impulsivity or dissociation), and risk factors (e.g., childhood trauma). (3) Investigating, if distinct NSS subdomains (e.g., integrative functions or complex motor behavior) will significantly differ between patients with BPD and those with SZ. (4) Finally, employing a descriptive and predictive linear discrimination analysis (LDA) to examine if both total NSS and subscale scores can discriminate between the three groups.

## Methods

### Participants

To reduce clinical and other phenotypic heterogeneity that has been frequently ascribed to gender by previous studies [25, 26], 108 female participants were included in this study, divided into three groups, i.e. 45 individuals with BPD, 30 individuals with SZ, and 32 HC. Recruitment of BPD and HC took place at the Department of General Psychiatry at Heidelberg University, Germany.

Participants in the BPD group met the DSM-5 criteria for BPD at the time of the study assessment. A prior BPD diagnosis was further confirmed by reviewing detailed medical records provided by the participants prior to being included in this study. Further inclusion criteria for BPD patients were age between 18 and 65 years and a stable psychotropic medication for at least 2 weeks. Exclusion criteria were a lifetime history of severe neurological or physical diseases that may influence brain functioning, any severe DSM-5 substance-use disorder fulfilling DSM-5 diagnostic criteria (except for tobacco) in the past 6 months, and a history of a psychotic disorder (particularly schizophrenia-spectrum disorders, major depressive or bipolar disorder with psychotic features, or psychotic disorders due to psychotropic drug use or due to a medical condition). Of note, other mental disorders, such as, e.g., major depressive disorder or post-traumatic stress disorder (PTSD), were not defined as exclusion criteria, since they are regarded as frequent comorbid disorders in BPD, and since we considered the omission of such comorbidities as not representative of the clinical picture that dominates psychiatric practice [27]. In this sample, current and lifetime comorbid mental disorders included major depressive disorder, posttraumatic stress disorder, anxiety disorder, specific phobia, social phobia, obsessive-compulsive disorder, attention–deficit/hyperactivity disorder, and eating disorders. One participant also had a lifetime history of amphetamine use disorder, being in full remission at the time of being included in this study.

All BPD patients were medicated, with antidepressants being the most prevalent drug class, followed by second-generation antipsychotics, mood stabilizers, and stimulants. None of the patients received benzodiazepines.

In BPD patients, antidepressant and antipsychotic medication were standardized as Imipramine [IMI] [28] and Olanzapine [OLZ] [29] equivalents, respectively. IMI and OLZ equivalents were z-transformed, summed up, and included as covariates in subsequent analyses (see below).

Patients with SZ according to ICD-10 (F20.x) were recruited at the Department of Psychiatry and Psychotherapy at the Central Institute of Mental Health in Mannheim, Germany, and at the Department of General Psychiatry and Psychotherapy at Heidelberg University Hospital, Germany.

The diagnosis was confirmed using the German versions of the Structured Clinical Interview for DSM-IV-TR axis I and II disorders (SCID) and examination of the detailed medical report. Further inclusion and exclusion criteria were equal to the BPD group (age 18–65, stable psychotropic medication for at least 2 weeks, no lifetime history of severe neurological or physical diseases that may influence brain functioning, and no severe DSM-5 substance-use disorder (except for tobacco) in the past 6 months).

All SZ patients were medicated with second generation antipsychotics, either in monotherapy or in combination. We evaluated the antipsychotic medication using OLZ equivalents [29]. None of the patients received benzodiazepines. HC were recruited via personal communication and community advertisements. The inclusion criteria for HC were the absence of a personal or family history of any mental disorder, as well as the absence of a personal history of neurological disorders or other physical diseases that could potentially influence brain functioning.

The study was conducted in accordance with the Declaration of Helsinki. Ethical approval was received from the local ethics committee (Medical Faculty Heidelberg at Heidelberg University, Germany). Written informed consent was obtained from all participants after a detailed explanation of the aims and procedures of the study.

### Clinical assessment

NSS were assessed by the well-established Heidelberg NSS scale (HNSS) [1], which contains 16 items that tap into five functional subdomains [1]. Motor coordination (MoCo), as assessed by five items (Ozeretski’s test, diadochokinesia, pronation/supination, finger-to-thumb opposition, speech articulation); [2] integrative functions (IF), as assessed by three items (standing and gait, tandem walking, two-point discrimination); [3] complex motor tasks (CoMT), as assessed by two items (finger-to-nose and fist-edge-palm); [4] right/left and spatial orientation (RLSpO), as assessed by four items (right/left orientation, graphesthesia, face-hand test, stereognosis); [5] hard signs (HS), as assessed by two items (arm holding test, mirror movements). All items are rated on a 0- to 3-point scale (absent, slight, present, marked). HNSS shows good internal reliability (Cronbach’s α 0.83) and a good interrater reliability (0.88) [1]. Assessment of the NSS was performed by trained raters. The raters were not blind to the diagnoses of the participants.

In BPD patients, depressive symptoms were assessed with the Hamilton depression scale with 21 items (HAMD-21) [30]. Childhood trauma was assessed with the self-rated Childhood Trauma Questionnaire (CTQ) [31–33], which includes subscores measuring emotional abuse, physical abuse, sexual abuse, emotional neglect, and physical neglect. Impulsivity was assessed with the self-rated Barrett Impulsiveness Scale-11 (BIS-11) [34]. In further analyses of the BIS-11, the total score as well as two subscores (cognitive and behavioral) were considered according to a more recent two-factor solution [35]. The severity of borderline symptoms was self-rated using a short version of the borderline symptom list (BSL-23) [36]. Dissociative symptoms were self-rated using the Dissociation Tension Scale (DTS) [37, 38].

In SZ patients, the Positive and Negative Syndrome Scale (PANSS) [39] and the Brief Psychiatric Rating Scale (BPRS) [40] were used to assess positive, negative, and overall symptom load.

### Data analysis

Data analysis was performed using the statistic software R 4.2.2 [41] with the following additional packages: *ppcor* for partial correlation [42], *MASS* for LDA [43] *caret* for prediction of the LDA [44], *agricolae* for Fisher LSD-test [45], *ggplot2* for graphs (Boxplots, correlation graphs) [46].

Between-group differences (BPD vs. HC and BPD vs. SZ) in the demographic data were calculated using the Wilcoxon rank sum test (as they did not meet the criteria for a t-test or Welch test, which was previously tested with the Shapiro-Wilk test (normality) and Levene test (homogeneity of variance). For visualization of the distribution of the HNSS subscores, boxplots were used.

Multivariate analysis of variance (MANOVA) was performed with all three groups (BPD, SZ, and HC) and HNSS subscores. A post hoc test LDA following the workflow of Andy Field [47] was performed, using group as the grouping variable and NSS subscores as an independent variable. The LDA analysis was followed up by a calculation of the predictive values of the LDA model. Additionally, as a post hoc test, Fisher’s LSD test was applied, looking at the group differences for each HNSS subscore separately. *P*-values < 0.05 were considered significant.

Within the BPD sample, being the diagnostic group of major interest in this study, partial Spearman correlations were performed using age and medication (as described above) as covariates for total HNSS and its subscores and HAMD-21, BIS-11 (total and cognitive and behavioral subscore), DTS, CTQ (total and its subscores emotional abuse, physical abuse, sexual abuse, emotional neglect, and physical neglect), and BSL-23. Significant partial Spearman correlations were visualized as scatter plots. *P*-values < 0.05 were considered significant.

## Results

### Demographics and clinical scores

BPD and HC did not differ significantly in age and education years. BPD and HC differed significantly in HAMD-21, DTS, CTQ, BIS-11, and BSL-23. BPD and SZ differed significantly in age and education (see Table 1 for more details).

**Table 1:** Demographics

		HC (*n* = 32)		BPD (*n* = 45)		SZ (*n* = 30)			
		**Mean**	**SD**	**Mean**	**SD**	**Mean**	**SD**	*p*-value (BPD vs. HC)	*p*-value (BPD vs. SZ)
Age (years)		24.3	5.2	27.8	9.1	34.4	7.3	0.127	**0.005**
Education years		15.9	2.6	15.3	3.0	13.7	3.3	0.256	**0.030**
HAMD-21		0.5	1.1	10.3	7.8	n.a.		**< 0.001**	
DTS		18.4	24.0	368	282.3	n.a.		**< 0.001**	
CTQ		43.9	9.3	68.5	16.8	n.a.		**< 0.001**	
BIS-11		54.6	6.9	72.3	10.4	39.8	38.9	**< 0.001**	
BSL-23		2.8	2.9	43.3	20.1	n.a.		**< 0.001**	
PANSS positive						13.9	7.0		
PANSS negative						14.5	8.5		
PANSS general						31.0	10.3		
PANSS total						59.4	22.5		
BPRS total						32.9	12.2		
HNSS total score		2.4	1.8	10.2	7.8	16.8	8.3	**< 0.001**	**< 0.001**
HNSS motor coordination (MoCo)		0.9	1.2	3.7	3.5	6.4	4.6	**< 0.001**	**0.006**
HNSS integrative functions (IF)		0.3	0.6	2.1	1.8	2.5	1.8	**< 0.001**	0.250
HNSS complex motor tasks (CoMT)		0.1	0.4	1.1	1.3	2.8	2.1	**< 0.001**	**< 0.001**
HNSS right/left & spatial orientation (RLSpO)		0.8	1.0	1.7	2.8	2.5	2.1	0.210	**0.015**
HNSS hard signs (HS)		0.5	0.8	1.5	1.3	2.6	1.8	**< 0.001**	**0.002**

Wilcoxon rank sum test. Significant results in bold font

Abbreviations: BIS-11, Barratt Impulsiveness Scale-11: BPD, borderline personality disorder; BPRS, Brief Psychiatric Rating Scale; BSL-23, short version of borderline symptom list; CoMT, complex motor tasks; CTQ, Childhood Trauma Questionnaire; DTS, Dissociation Tension Scale; HAMD-21, Hamilton depression scale; HC, healthy control; HNSS, Heidelberg Neurological soft signs scale; HS, hard signs; IF, integrative functions; MoCo, motor coordination; n.a. not available; NSS, Neurological soft signs; PANSS, Positive and Negative Syndrome Scale; RLSpO, right/left and spatial orientation; SZ, schizophrenia

### NSS: comparisons between BPD, SZ and HC

**Fig. 1:**
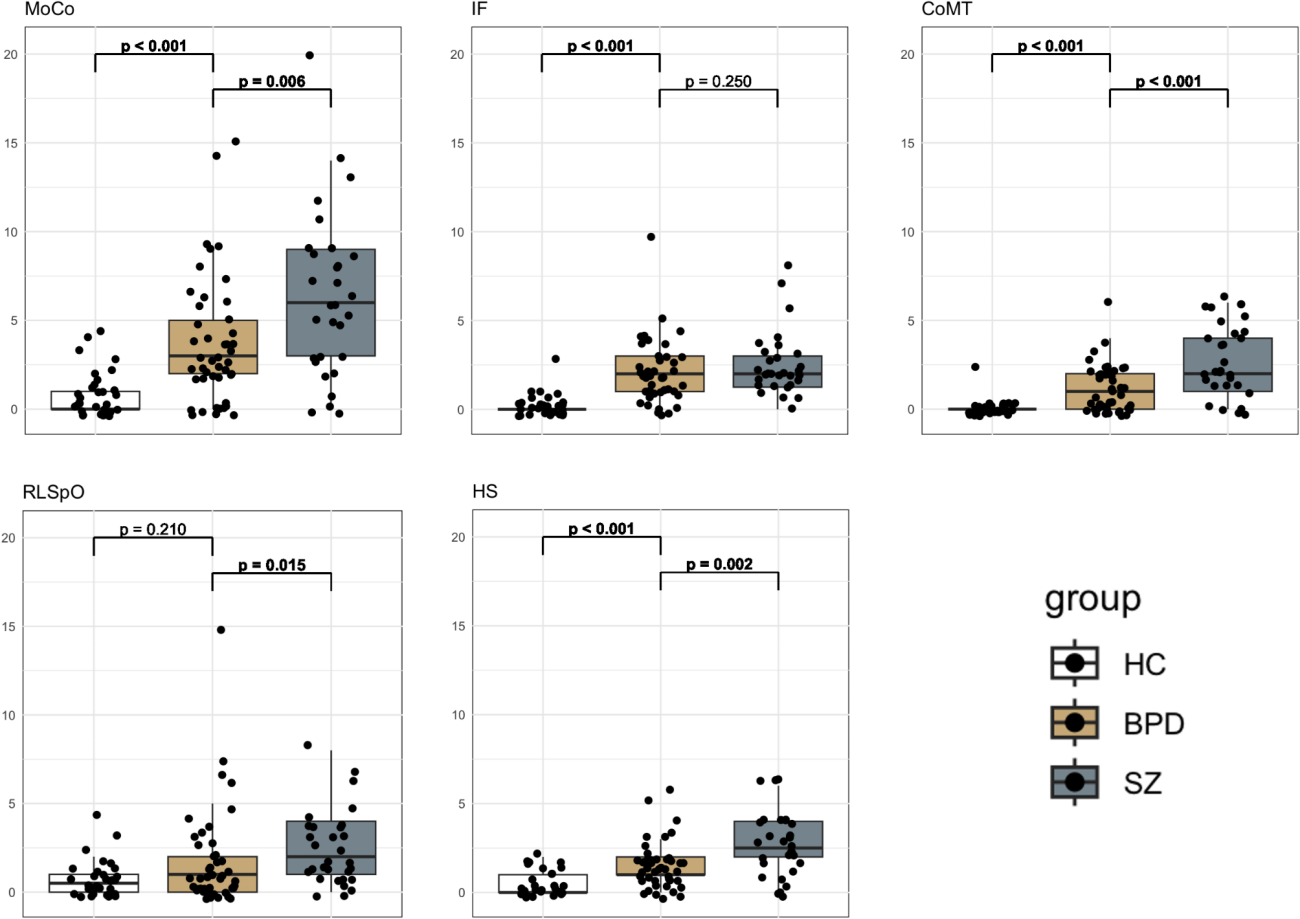
Comparison of HNSS subscores (MoCo, IF, CoMT, RLSpO, HS) between HC, BPD, and BPD and SZ. *P*-values of the Wilcoxon rank sum test. Significant *p*-values are in bold font. Abbreviations: CoMT, complex motor tasks; HC, healthy control; HNSS, Heidelberg Neurological soft signs scale; HS, hard signs; IF, integrative functions; MoCo, motor coordination; RLSpO, right/left and spatial orientation; SZ, schizophrenia

BPD had significantly greater NSS scores in the subscales MoCo, IF, CoMT, and HS compared to HC (Fig. 1). SZ exhibited significantly higher NSS in the MoCo, CoMT, RLSpO, and HS subscales compared to BPD.

**Fig. 2:**
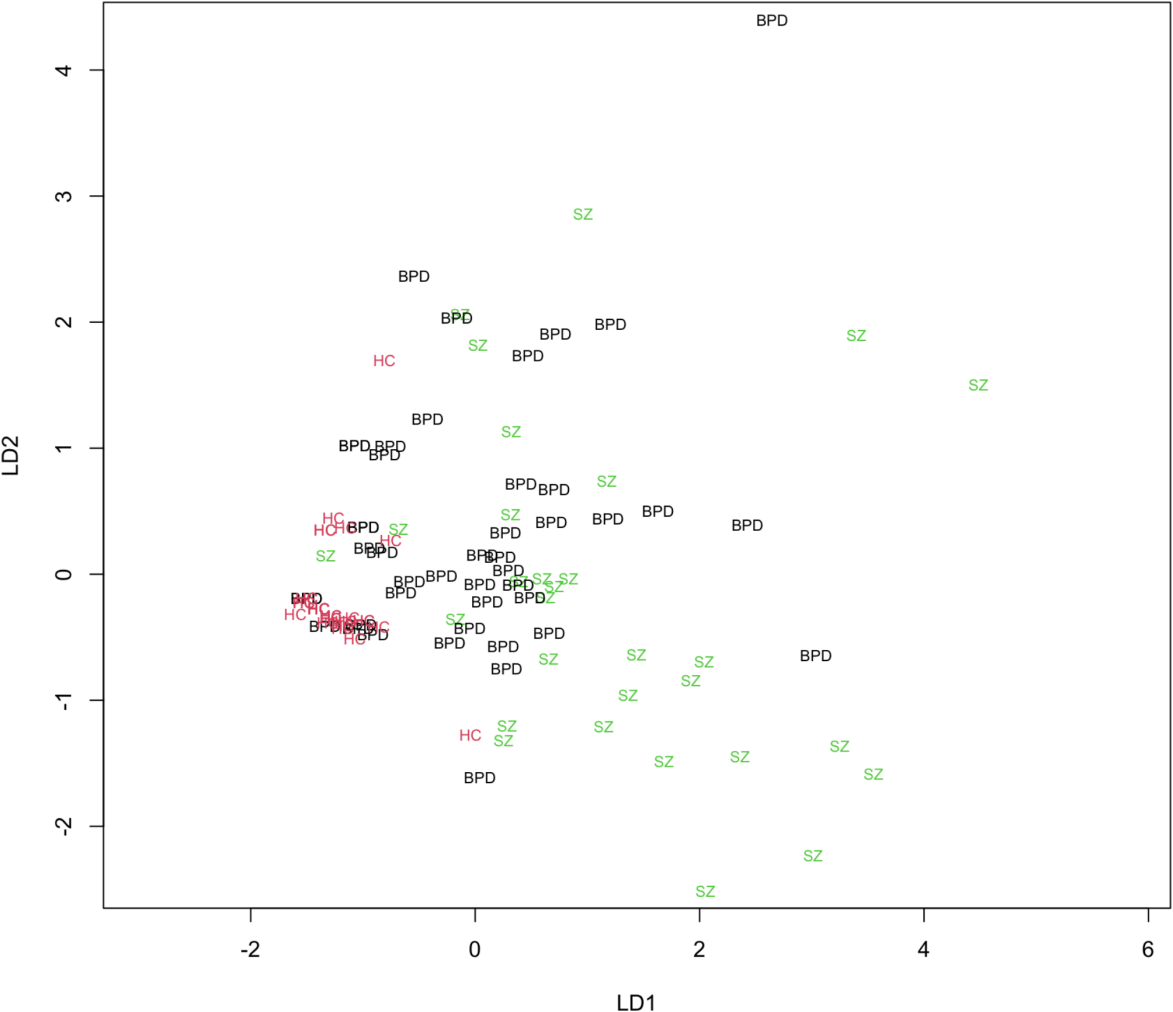
Linear discriminant analysis between BPD (black), SZ (green), and HC (red). Abbreviations: BPD, borderline personality disorder; HC, healthy control; LD, linear discriminant; SZ, schizophrenia

MANOVA showed significant group differences between HC, BPD and SZ (*p* < 0.001). MANOVA was followed by LDA and a post hoc test that revealed two discriminant functions. The first explained 91.95% of the variance, the second 8.05%. The coefficients of the first discriminant functions were as follows: MoCo 0.026, IF -0.239, CoMT − 0.460, RLSpO − 0.107, and HS -0.268. The second discriminant function had the following coefficients: MoCo − 0.031, IF 0.639, CoMT − 0.367, RLSpO − 0.079, and HS -0.073. The discriminant function plot showed that the first variable differentiated between HC and the two patients’ groups (BPD and SZ), whereas the second function could not differentiate between the groups (see Fig. 2).

Additionally, looking at predictive LDAs, the discriminate function had 72.0% accuracy, looking at the three groups, for BPD the sensitivity was 75.6% and the specificity 71.0%, for SZ the sensitivity was 46.7% and the specificity 94.8% and for HC the sensitivity was 90.6% and the specificity 89.3%.

The Fisher’s LSD post hoc test revealed differences between all three groups in MoCo, CoMT, and HS. For IF only HC was different compared to SZ and BPD, for RLSpO only SZ and HC were different, and BPD did not differ significantly from SZ or HC.

### Partial spearman correlations between NSS and clinical variables in BPD

**Fig. 3:**
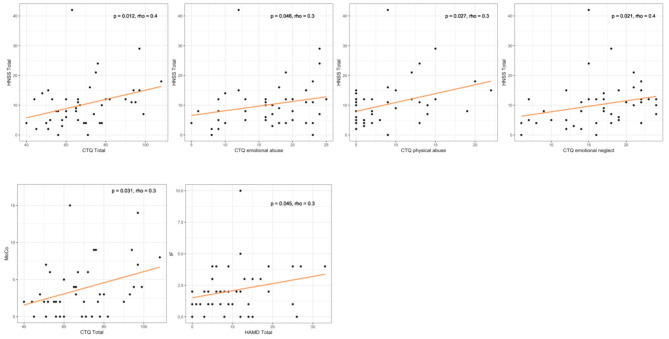
Visualization of significant partial Spearman correlations between HNSS and scores in BPD-group with scatter plots. There were no significant partial correlations with BSL-23, BIS-11 (total and subscores), or DTS. Abbreviations: BIS-11, Barratt Impulsiveness Scale-11: BPD, borderline personality disorder; BSL-23, short version of borderline symptom list; CoMT, complex motor tasks; CTQ, Childhood Trauma Questionnaire; DTS, Dissociation Tension Scale; HAMD-21, Hamilton depression scale; HC, healthy control; HNSS, Heidelberg Neurological soft signs scale; HS, hard signs; IF, integrative functions; MoCo, motor coordination; NSS, Neurological soft signs; RLSpO, right/left and spatial orientation

There were significant positive partial correlations between HNSS and CTQ Total scores. In particular, we found a significant association between CTQ Total, HNSS Total and CTQ emotional abuse, HNSS Total and CTQ physical abuse, and HNSS Total and CTQ emotional neglect (Fig. 3). In the HNSS subscores there were significant partial correlations between MoCo and CTQ Total, and IF and HAMD Total (Fig. 2). There were no significant partial correlations with BSL-23, BIS-11 (total and subscores), or DTS.

## Discussion

To the best of our knowledge, this is the first study that compared NSS levels between BPD and SZ. This study was designed (i) to investigate if there are significant differences in the NSS levels between BPD patients and HC, especially on the subscales MoCo and IF, (ii) to examine the relationships between NSS and specific symptom clusters (e.g., impulsiveness, dissociative symptoms, and depressive symptoms), experiences of childhood trauma and overall symptom severity in BPD, and (iii) to investigate if distinct NSS subscales can specifically classify participants with BPD and SZ. We will now discuss these findings in more detail.

First, participants with BPD had higher NSS scores than those with HC. Differences were found in the NSS total score as well as in MoCo, IF, CoMT, and HS subscales scores. These findings are in line with previous studies [19, 21–24], albeit a more direct comparability is limited due to the nonuniform use of the NSS assessments and different items within individual NSS subscales in previous research. In terms of NSS subscores, we were able to confirm our prior hypothesis and support previous evidence of higher IF and MoCo scores in BPD versus HC. Interestingly, this study did not discover any associations between NSS and psychopathological symptoms such as borderline-specific symptoms, impulsivity, and dissociative symptoms. These findings differ partially from those of previous studies. For instance, Khoweiled et al. found a significant correlations between NSS and borderline-specific symptom severity (measured with the Borderline Personality Questionnaire, a self-questionnaire with 80 items with dichotomous response) and impulsivity (measured by the BIS-11) [23]. To the authors’ knowledge, only one other study has explored the potential factors influencing NSS in BPD up to this point: In a combined correlation analysis involving both the BPD and HC groups, Arbabi et al. identified significant correlations with depressive symptoms, anxiety, and impulsivity [19]. However, it is important to note that there were also observable group differences in all three scales, thus potentially increasing the risk of false positive findings.

Second, the NSS total score and the MoCo subscore showed significant positive correlations with the CTQ, where both the HNSS total and the “motor coordination” scores converged on the CTQ total and the physical abuse scores. There are several potential mechanisms that may explain the association between NSS and CTQ scores in BPD patients: Childhood trauma is already well-studied as an important risk factor for the development of BPD [48]. In this regard, it is worth noting that NSS are physiological in children, and that such sensorimotor signs disappear gradually as a consequence of brain maturation during childhood and adolescence [4, 5]. In BPD this process seems to be disrupted. The association between NSS and childhood trauma suggests that traumatic childhood events may interfere with normal sensorimotor development, i.e., putatively disrupt the physiological sensorimotor maturation process. Trauma-related alterations in neural pathways and structures may lead to the development of NSS in individuals with BPD. In this regard, the findings of this study support a “two-hit” model of BPD, where childhood trauma can disrupt developmental processes far beyond affective and cognitive processing. Gene-environment interactions can be clearly associated with alterations in brain structure and function [49, 50] as well as psychosocial conditions [51]. The emotional and psychological distress resulting from childhood trauma and the aberrant neurodevelopment as reflected by NSS may contribute to the development and severity of BPD-associated psychopathological symptoms. In addition, it is well established that childhood trauma predicts disease course and treatment outcome in BPD [52, 53]. Adding sensorimotor signs, particularly NSS, as an objective proxy for past trauma may refine existing outcome prediction models [54]. Eventually, it is important to note that the precise mechanisms underlying the association between NSS and CTQ scores in BPD are not fully understood, and further research is needed to explore these relationships in greater detail.

Third, a positive correlation between IF and depressive symptoms was found. This finding is consistent with studies that have found higher NSS scores in mood disorders such as major depressive disorder (MDD) [16] and bipolar disorders [55]. However, the association between NSS and depressive symptoms in BPD is a complex and multifaceted phenomenon. A possible explanation might be that depressive symptoms can sometimes go along with sensorimotor alterations, although these alterations are not typically considered diagnostic criteria for MDD. Furthermore, the link between NSS and depressive symptoms again emphasizes a transdiagnostic connection between sensorimotor and affective domains in BPD [56].

Fourth, while participants with SZ exhibited notably higher scores in the NSS subscales MoCo, CoMT, RLSpO, and HS than those with BPD, a post hoc analysis comparing all three groups revealed a significant distinction between the HC and both the BPD and SZ groups. Our findings of increased NSS levels in SZ are in line with previous studies (for a meta-analysis, see [9]). However, our study is the first to compare NSS in BPD with NSS in SZ. Participants with BPD showed similar NSS patterns compared with participants with SZ, especially in the IF subscale, which may indicate an overlap of the sensorimotor phenomena of BPD and SZ. There might be similar developmental aberrations during adolescence regarding the maturation of the sensorimotor networks in BPD and SZ or even a similar vulnerability for the disruption of the sensorimotor maturation process, although this needs to be addressed by further research more directly. Although childhood trauma is a risk factor for the development of SZ [57], we did not identify any studies showing that childhood trauma may explain higher levels of NSS in SZ compared to HC. However, an earlier study by Gurvits et al. [58] found that NSS are present in both women and men with PTSD who have experienced various types of traumatic events as children and adults. Furthermore, the study by Zhao et al. [59] found that depressive patients experiencing childhood emotional or physical neglect showed more frontal area-related NSS compared to their respective group without maltreatment. Although this study cannot provide information on the direct association between childhood trauma and SZ, it provides evidence that child maltreatment can contribute to higher NSS in psychiatric disorders.

The strengths of this study are the larger sample size of patients with BPD compared to previous studies on NSS in BPD, its transdiagnostic design, and the different statistical methods used. However, this study also has some limitations: One of the major potential limitations of this study is its cross-sectional design, as the degrees of NSS in BPD could potentially vary over time. At present, longitudinal studies investigating NSS in BPD are not available, and therefore this prominent question needs to be addressed by future research. Although our results show that the NSS are independent of the acute psychopathological symptoms, we cannot make any statement on the temporal stability of the NSS based on this cross-sectional study. Therefore, we strongly endorse longitudinal study designs that examine the temporal stability of NSS. We also acknowledge that HNNS raters were not blind to patient’s diagnoses. Yet, in this regard it is noteworthy that a previous meta-analysis that considered patients with SZ and healthy individuals at risk for SZ did not reveal significant effects of rater blinding Neelam et al. (2011). Also, while we show that the degree of soft sign load significantly differs between BPD and SZ patients, the LDA did not significantly discriminate between the patient groups [60]. We acknowledge the extensive number of statistical tests conducted in this study and recognize that the findings presented and discussed were not corrected for multiple comparisons. Considering the limited research on the sensorimotor domain in BPD and the current lack of transdiagnostic studies comparing individuals with BPD to other diagnostic groups, we regard this study as predominantly exploratory. These findings should therefore be interpreted with caution and await further independent replication and extension. Another potential limitation to consider is that, apart from severe substance-use disorder in the past six months and current or lifetime psychotic disorders, no other psychiatric comorbidities were excluded in the BPD group. Yet, psychiatric comorbidities in BPD are far from being an exception (e.g., the prevalence for mood disorders has been estimated at up to 96%) [27], so excluding patients with psychiatric comorbidities would likely lead to an unrepresentative study cohort. Finally, we are aware of the fact that although the CTQ has good test-retest reliability for shorter periods (< 1 year) [33, 61], there are no studies available that provide robust evidence for good test-retest reliability for longer periods. Also, the recall of memories is affected as such in patients with psychiatric disorders, who recall fewer specific and more general memories [62]. While childhood trauma clearly has a crucial role in the neurodevelopment, disease course, and outcome of BPD, assessing childhood trauma nevertheless is prone to inaccuracies and as such, some of these inaccuracies may also have an impact on the associations between CTQ and NSS, as shown by this study. Additionally, the use of the CTQ included childhood experiences that do not traditionally align with the definition of traumatic events but have been shown to be particularly relevant in BPD. The meta-analysis of Porter et al. (2020) identified emotional abuse and neglect as the most common childhood adversity in individuals with BPD compared to healthy controls [63].

## Conclusion

In conclusion, our comprehensive study investigating NSS in BPD and SZ revealed novel insights into these complex mental disorders. By identifying and comparing the levels of NSS, we enhanced our understanding of the distinct and shared sensorimotor profiles in both disorders. These findings underscore the importance of a multi-dimensional approach to psychiatric research and hold the potential to inform more targeted interventions and personalized treatment strategies for individuals grappling with BPD and SZ based on the sensorimotor domain.

## Data Availability

Data will be made available on scientifically reasonable request.

## Abbreviations

AVH: Auditory verbal hallucinations
BIS-11: Barratt Impulsiveness Scale-11
BPD: Borderline personality disorder
BPRS: Brief Psychiatric Rating Scale
BSL-23: Short version of borderline symptom list
CoMT: Complex motor tasks
CTQ: Childhood Trauma Questionnaire
DTS: Dissociation Tension Scale
HAMD-21: Hamilton depression scale
HC: Healthy control
HNSS: Heidelberg NSS scale
